# Association between Mild Overweight and Survival: A Study of an Exceptionally Long-Lived Population in the Sardinian Blue Zone

**DOI:** 10.3390/jcm13175322

**Published:** 2024-09-09

**Authors:** Giovanni Mario Pes, Alessandra Errigo, Maria Pina Dore

**Affiliations:** 1 Dipartimento di Medicina, Chirurgia e Farmacia, University of Sassari, Clinica Medica, Viale San Pietro 8, 07100 Sassari, Italy; a.errigo@studenti.uniss.it (A.E.); mpdore@uniss.it (M.P.D.); 2 Sardinia Blue Zone Longevity Observatory, 08040 Santa Maria Navarrese, Italy; 3 Department of Medicine, Baylor College of Medicine, One Baylor Plaza, Houston, TX 77030, USA

**Keywords:** body mass index, weight excess, obesity paradox, longevity, Sardinia, elderly people

## Abstract

**Background/Objectives**: Overweight and obesity are generally considered risk factors for premature mortality. However, scientific evidence suggests that among older populations, mild conditions of overweight might be associated with reduced comorbidity and longer survival. This study investigates the potential association between anthropometric parameters and survival among a cohort of nonagenarians in Sardinia, Italy. **Methods**: This study included 200 subjects (50% females) aged 89 and older, enrolled in 2018 in the Sardinian Blue Zone—a population known for longevity—and followed for up to six years. Anthropometric variables such as body height, weight, age, sex, comorbidity, disability, and food group intake were collected using validated questionnaires and analyzed through multivariable analysis. **Results**: Out of 200 participants at baseline, 28 (14%) were still alive after six years of follow-up (females 10%, males 18%). Mean survival was 3.36 years (range 0.1–6.9 years) for males and 3.03 years (range 0.2–6.6 years) for females. Participants with a Body Mass Index (BMI) in the range of 25.0–27.0 kg/m^2^ among males and 25.0–27.2 kg/m^2^ among females had longer survival compared to those who were underweight (*p* = 0.002) or obese (*p* < 0.0001). The Cox proportional hazards regression model, adjusted for age, sex, and comorbidity, revealed a statistically significant association between the BMI and survival, demonstrating an inverted–U relationship. This indicates that mild overweight was associated with a survival advantage compared to both normal weight and obesity. **Conclusions**: Our study indicates that mild, but not severe, overweight in nonagenarians is associated with extended lifespan. Therefore, primary care physicians and geriatricians should exercise caution before recommending calorie-restricted diets for mildly overweight elderly patients.

## 1. Introduction

Overweight can be defined as an excessive accumulation of adipose tissue, typically indicated by a body mass index (BMI) of 25.0–29.9 kg/m^2^, which is generally associated with increased morbidity and mortality risk [1]. In resource-rich countries, the burden of overweight is growing in terms of healthcare costs due to the rising number of individuals affected by overweight-related chronic illnesses who require frequent hospitalizations, extensive medical treatments, and invasive surgical procedures [2]. Overweight prevalence is increasing, particularly among the oldest age groups, although this varies widely by ethnicity. For instance, Hajek et al. reported a prevalence of overweight and obesity of 37.9% and 10.2%, respectively, in a multicenter prospective cohort study of individuals aged 85 years and older [3]. Similarly, Dinu et al. found that 33% of individuals aged 90 and older in central Italy were overweight [4]. In contrast, Tarui et al. reported a lower prevalence of overweight (<25%) in individuals over age 80 in Japan [5].

Although overweight in midlife is associated with an increased risk of several chronic diseases and consequently, premature mortality [6], a number of observational studies conducted in the past decade in various ethnic populations have suggested that a mildly increased BMI in the elderly may be associated with extended survival [7,8,9,10]. This association of being overweight with less comorbidity and increased longevity has often been referred to as the “overweight paradox” or “BMI paradox” [11], and a recent Mendelian randomization study has provided robust evidence that overweight is causally linked to a longer lifespan [12]. Moreover, in the elderly population, the condition of overweight/obesity is often associated with a general decrease in free fat mass, a phenomenon usually referred to as “sarcopenic obesity” [13]. In fact, in the elderly, excess weight and malnutrition can coexist and can be detected using specific inventory tests such as the Mini-Nutritional Assessment (MNA) [14].

Analyzing the relationship between overweight and longevity is particularly relevant in populations with a significant proportion of individuals who survive into extreme old age. One such population is found on the Mediterranean island of Sardinia, Italy, known as a “*Blue Zone*” [15]. Previous studies conducted by our research team on nonagenarians in this area highlighted that 29.5% of males and 33.7% of females were overweight. In comparison, the percentage of obesity was lower, at 14% and 6% in males and females, respectively [16,17]. Such a relatively high proportion of excess weight in a population renowned for its longevity may seem unexpected and warrant further investigation.

The main objectives of the present study were to investigate (i) the relationship between overweight and survival in the oldest age group from this well-known longevity area and (ii) the potential impact of dietary habits on weight gain at such an advanced age.

## 2. Materials and Methods

### 2.1. Study Design

This was a retrospective longitudinal study conducted on a sample of nonagenarians recruited between 2018 [18] and 2023 [19] in the “*Blue Zone*” area of central Sardinia, where a population known for its longevity has been identified. The study protocol was approved by the institutional review board of the local *Comitato Etico ASL no. 1 di Sassari,* Italy, protocol no. 2101/CE. Written informed consent was obtained from all participants or their caregivers in cases of severe dementia.

### 2.2. Patient Eligibility

Details of the analyzed cohort have been described previously [17,18]. Inclusion criteria were Sardinian origin (with at least four grandparents born within the *Blue Zone*) and age over 89 years. Data on demographics, diet, and lifestyle were collected during a geriatric multidimensional evaluation using validated questionnaires (see details below). Exclusion criteria included unavailability of any of the following: body weight and height, comorbidity, and basic and instrumental activities of daily living.

### 2.3. Data Collection

All participants underwent anthropometric measurements by trained operators using standardized procedures [20]. Briefly, body height was measured with a portable stadiometer, aligning the patient’s head according to the Frankfurt horizontal plane, and recorded to the nearest centimeter. Body weight was measured with an electronic scale with a precision of up to 0.1 kg. The BMI was calculated as weight/height^2^ (kg/m^2^). To better characterize overweight, the BMI range was divided into two equally sized subgroups, separately in males and females, using a median split. More specifically, a BMI between 25.0 and 27.0 kg/m^2^ for males and between 25.0 and 27.2 kg/m^2^ for females was considered mild overweight, and between 27.1 (males) or 27.3 (females) and 29.9 kg/m^2^ as severe overweight. Since most participants had provided their approximate weight at age 60, this variable was also included in the analysis. Additionally, waist circumference, average calf circumference (measured in the gastrocnemius muscle), average brachial circumference (measured in the biceps muscle), and average leg length were measured in centimeters according to established techniques [21].

### 2.4. Assessment of Dietary Patterns

Participants were asked about their dietary intake using a validated qualitative 16-item food frequency questionnaire (FFQ) [16,19,22] covering the following foods: *cereals* (bread, pasta); *vegetables* (legumes, leafy greens, and fruit); *tubers* (potatoes); *meat* (beef/pork, sheep/goat, chicken); *seafood* (mainly fish); *dairy products* (milk, cheese); *fats* (olive oil, lard); *sweets* (mainly traditional homemade cookies); *coffee* and *wine*. Food frequency was coded into five categories: never/rarely, 2–3 servings/month, 1–2 servings/week, 3–5 servings/week, and every day, at the usual serving size. Food frequency was ranked into five categories: never/rarely (rank 1), 2–3 servings/month (rank 2), 1–2 servings/week (rank 3), 3–5 servings/week (rank 4), every day (rank 5).

### 2.5. Covariates

A number of covariates were gathered from participants’ baseline questionnaire: sex, age at enrollment, smoking status, physical activity, and disability. Smoking status was categorized as never smoker, former smoker, and current smoker. For physical activity, participants were asked whether they engaged in physical activity and how many times per week, categorized as <3 times/week or ≥3 times/week. Functional independence was measured using the Katz scale, which assesses difficulty in performing basic activities of daily living (ADLs) and instrumental activities of daily living (IADLs) [23]. The ADL scale was assigned a score ranging from zero (complete dependence) to six (complete independence) for each independent function. The ADLs included dressing without help, moving around the room, eating, bathing, using the bathroom, and rising from a bed or chair. Independence in physically and cognitively more demanding activities was evaluated by the IADLs, which include activities common in Latin countries, such as managing one’s own money, using the telephone, using transportation, and taking medications [24]. The Cumulative Illness Rating Scale (CIRS) was used to estimate comorbidity [25].

### 2.6. Statistical Analysis

Data from anthropometric measurements and questionnaires were summarized using descriptive statistical indicators such as mean and standard deviation (SD) for the continuous variables and absolute and relative frequencies, or ranks, for categorical variables. The normality of continuous variables was assessed using the Kolmogorov–Smirnov test and Q–Q plots prior to analysis. Variables meeting normality criteria were expressed as the mean ± SD, while non-normally distributed variables were presented as the median (interquartile range). Continuous variables with skewed distributions were log-transformed. The statistical significance of differences among BMI groups was tested by one-way ANOVA, combined with Tukey’s post hoc analysis. For m × n contingency tables, analysis was performed using the χ^2^ test or Fisher’s exact test. Ranks were considered as ordinal variables, and the comparison between subgroups was performed using the Mann–Whitney U test for independent samples or the Kruskall–Wallis test.

Age at death was calculated from dates of birth and death. For participants who were still alive at the end of the follow-up, the ages at censoring were determined based on the date of birth and the last follow-up. The BMI was age-adjusted before statistical tests. The association between anthropometric variables and survival was estimated using the Kaplan–Meier curves and the log-rank test, as well as Cox proportional hazards regression models. The hazard ratio (HR) and corresponding 95% confidence interval (CI) were calculated for the association of independent variables with survival, comparing the highest to the lowest BMI ranges. The proportional hazards assumptions and model fitness were verified using the Wald test, which indicated that the assumptions were satisfied. All statistical analyses were performed with SPSS for Windows (version 22.0, Chicago, IL, USA). Odds ratios (ORs) and their 95% Cis were calculated by exponentiating the regression coefficients. A two-sided threshold value of *p* < 0.05 was considered statistically significant.

## 3. Results

### 3.1. Basic Demography

Table 1 shows the characteristics of 200 community-dwelling older adults (50% females) who participated in the study.

At the end of the 6-year follow-up period, 28 participants were still alive (18 males and 10 females). The duration of follow-up for those who died during the follow-up was 2.83 ± 1.56 years and 2.79 ± 1.55 years in males and females, respectively.

### 3.2. Anthropometric Parameters

**Figure 1:**
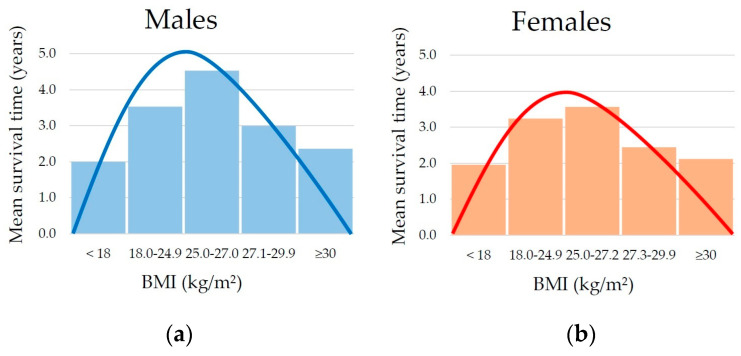
Mean survival time according to body mass index (BMI) categories in males (**a**) and females (**b**) showing a non-linear inverted–U relationship between BMI and longevity.

Of the 200 participants recruited between 2018 and 2023, only 28 were still alive in June 2024 (14%). Specifically, only one male and one female centenarian survived, with a mean survival of 3.36 ± 1.99 years for males and 3.03 ± 1.74 years for females. Figure 1 and Table 2 report the mean survival of subjects stratified according to BMI values at baseline. A survival peak significantly higher than that of underweight (*p* = 0.002) and obese (*p* = 0.044) individuals was observed, corresponding to the BMI range of 25.0–26.9 kg/m^2^.

**Figure 2:**
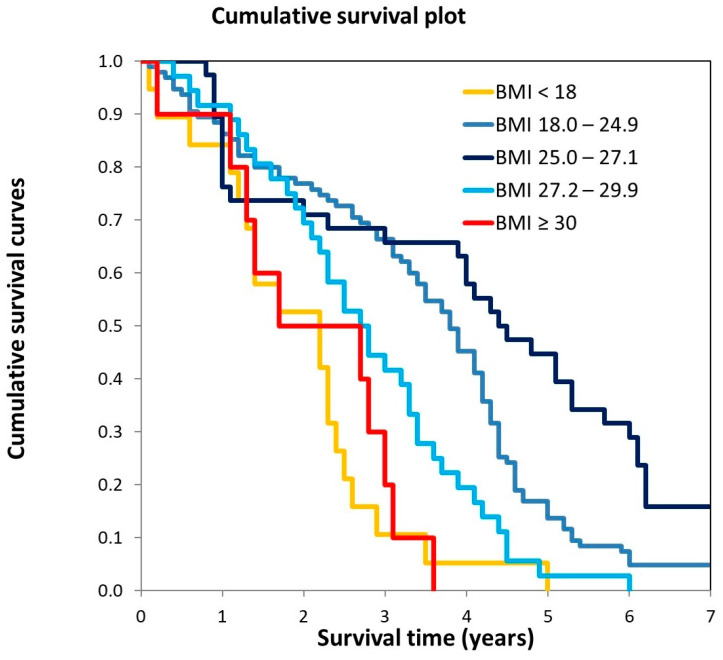
KM curves in participants stratified according to BMI at baseline. Pairwise log-rank test comparisons: BMI < 18 vs. BMI 25.0–27.1, *p* < 0.0001; BMI 18–24.9 vs. BMI 25.0–27.1, *p* = 0.016; BMI 25.0–27.1 vs. BMI 27.0–29.9, *p* = 0.003; BMI 25.0–27.1 vs. BMI ≥ 30, *p* = 0.001.

Figure 2 illustrates the Kaplan–Meier (KM) curves related to the survival of study participants, stratified by BMI value at recruitment.

It can be observed that survival was significantly longer among participants belonging to the BMI category of 25.0–27.1 kg/m^2^ (mild overweight), followed by those in the BMI range of 18.0–24.9 kg/m^2^ (normal weight) and 27.2–29.9 kg/m^2^ (severe overweight). Participants in the obese category showed the shortest survival. The log-rank test confirmed a statistically significant difference in survival between the mild overweight category and the other categories (Figure 2).

Table 3 reports the mean values of waist circumference, mean upper arm circumference, and calf circumference, taken as markers of lean mass, according to the categories of BMI at baseline.

No significant differences were observed between the mean values of waist and limb circumferences across BMI categories. Note that the highest values of lean mass corresponded to the categories of severe overweight and obesity.

### 3.3. Functional Independence

Statistically significant differences were observed for the ADL and IADL scores between the BMI 25.0–26.0 category and the BMI ≥ 30 category, while no significant difference was observed for the CIRS score category (Supplementary Table S2).

Table 4 reports the multivariable Cox proportional hazard model, with survival as the outcome variable. The categorical variable BMI at baseline (5 levels) was included as a predictor along with the covariates sex and age.

The analysis revealed a significant reduction in mortality for mildly overweight individuals compared to those in other BMI categories (HR 0.610, 95% CI 0.395–0.944). Among other covariates, only age at baseline showed a significant impact on survival.

### 3.4. Association of BMI at Baseline with Dietary Habits

Table 5 reports the consumption of food groups (expressed as ranks) in the subgroups of participants who died (n = 172) or who were still alive (n = 28) at the end of the follow-up period. Only fruit (*p* = 0.018) and olive oil consumption (*p* = 0.042) were significantly higher in the group of 28 participants who were alive at the six-years follow-up. The intake of all other foods was substantially similar in the two subgroups.

Table 6 reports the association between food consumption and BMI categories. The only significant differences were found for consumption of beef (χ^2^ = 23.30, *p* < 0.0001) and poultry meat (χ^2^ = 21.60, *p* < 0.0001) which was higher in overweight subjects (both mild and severe) compared to the other weight categories.

## 4. Discussion

Overweight, defined as the accumulation of body fat exceeding normal or desirable levels, has become increasingly common among older individuals, particularly those in resource-rich countries [26]. For many clinicians, this is a serious concern and a significant challenge for healthcare systems [27]. Numerous epidemiological studies have shown that in adults, overweight and obesity are important risk factors for potentially fatal disorders such as diabetes, coronary heart disease, stroke, and various types of malignancies. Despite this, evidence in the literature suggests that in advanced age, the relationship between overweight and illnesses tends to weaken progressively until it disappears. Interestingly, in some cases, a protective effect of overweight on overall health [28]—known as the “overweight paradox”—has been observed. Although some studies have reported better health status in older people with higher BMI [29], others did not confirm this association [30]. A systematic review by Dramé et al., examining 58 studies, found that only 28 studies showed more prolonged survival in patients with a BMI ≥ 25 kg/m^2^ [31]. However, a recent Mendelian randomization study has unequivocally demonstrated a causal relationship between overweight and longevity [12], suggesting that this counterintuitive association should change the attitude of geriatricians and practicing physicians when dealing with mild excess weight in the elderly.

The present study sheds light on this controversial concept using the model of long-lived populations, specifically in the central area of Sardinia [32]. The main finding reveals a non-linear relationship between BMI and survival among individuals aged ≥90 years in this community, followed up approximately six years. Specifically, survival was longer in mildly overweight participants, with a baseline BMI between 25.0 and 27.0 kg/m^2^ in males and between 25.0 and 27.2 in females, compared to those with normal or underweight BMI, as well as those with severe overweight (27.1–29.9 kg/m^2^) or obesity (≥30 kg/m^2^). This relationship, represented by an inverted U-shaped curve, was also observed when analyzing the self-reported BMI of participants at the age of 60, although only in relation to the difference between mild overweight and overt obesity.

The apparent protective effect of mild overweight on mortality, compared with both underweight and obese counterparts, cannot be attributed to a lower impact of sarcopenia, as age-adjusted arm and calf circumferences, indicators of sarcopenia, were not more remarkable in the mildly overweight group than in the more severely overweight groups. Additionally, limb circumferences were not significant predictors of survival. Statistical tests investigating the relationship between BMI and food consumption highlighted that beef and poultry meat intake were the main contributors to the mild increase in adipose tissue (both mild and severe overweight). Interestingly, bread, sweets, and wine consumption were not significantly associated with overweight. This could be explained by the fact that bread is a staple consumed regularly and in similar quantities by all elderly members of this community, resulting in negligible differences in adipose tissue accumulation in subgroups [33]. Additionally, sweets and wine were consumed minimally, contributing little to weight gain [16]. Historical data suggest that wine contributed only 3–4% to the daily energy intake of the Blue Zone diet [34]. Therefore, it can be concluded that in this population known for its exceptional longevity, a mild condition of overweight, partly favored by certain eating habits, appears to be a survival factor. At the same time, a BMI in the range of obesity appears to be detrimental, as previously reported in most adult populations.

The possible mechanisms underpinning the paradoxical association between adiposity and increased survival have been extensively discussed in the literature and are beyond the scope of this paper. Several potential explanations of the apparent protective effect of mildly increased BMI may be the following: (i) Worse health outcomes were observed in underweight older adults [35]. More specifically, underweight older adults could be malnourished, a condition known to increase the mortality risk [36], or already affected by chronic diseases or cancers, which are the actual cause of mortality. Unfortunately, most epidemiological studies do not specify whether underweight individuals have potentially fatal chronic diseases. In our study, type 2 diabetes was present in 15.8% of 38 subjects with severe overweight/obesity but only in 2.1% of 48 mildly overweight participants. (ii) Mildly overweight individuals could be less affected by sarcopenia than obese individuals, so their greater reserve of free fat mass would be the actual cause of the protective effect rather than the excess fat itself [37]. However, our study observed a lower prevalence of sarcopenia in the mildly overweight subgroup, as the average limb circumference, reflecting muscle mass, was more represented in the obese or severely overweight groups than in the mildly overweight groups, although the difference was not statistically significant. (iii) The oldest individuals are carriers of genetic variants conducive to increased survival. Such genetically favored individuals might have greater resistance to the harmful effects of being overweight, making mortality selection less effective. A similar phenomenon has been described in centenarians regarding their higher frequency of coagulation defects [38]. (iv) A slight excess of fat could protect the elderly from fractures in case of falls, as they may receive some protection by their extra padding, providing more efficient shock absorption [39]. (v) A mild pro-inflammatory effect of excess weight could trigger protective mechanisms that outweigh the negative effects of weight gain, according to the hormesis hypothesis [40]. (vi) Finally, regional fat distribution, infiltration of adipose tissue by inflammatory cells, the secretome profile of adipose tissue, and other molecular mechanisms may play a role in mediating the relationship between mild excess weight and positive outcomes [11].

The relatively high prevalence of overweight detected in the oldest members of the Sardinian Blue Zone population does not appear to be limited to this community. In the Costa Rican Blue Zone, the CRELES study reported a mean BMI of 26.87 kg/m^2^ [41], while in the long-lived population of Okinawa, Japan, the proportion of obesity ranged from 10 to 40% [42], suggesting an association between exceptional longevity and mild overweight.

On the other hand, research on calorie restriction has provided indisputable evidence that it extends both lifespan and health benefits in adults. The CALERIE study has clearly highlighted the benefits of this approach [43,44]. Similar studies on the island of Okinawa, another long-lived population, have established a strong link between caloric restriction and longevity [45].

It has long been known that from a cardiometabolic point of view, obesity is a heterogeneous entity [46]. For example, the most clinically significant heterogeneity is linked to the body distribution of adipose tissue, e.g., visceral vs. subcutaneous. It could not be excluded that such heterogeneity may also extend to overweight, albeit to a more nuanced extent. In agreement with other studies, we also observed that only mildly overweight participants had a survival advantage.

Several limitations must be noted. The relatively small sample size is partly due to the secluded, rural nature of the area, where older people tend to be overprotected by their families and often refuse comprehensive geriatric assessment during a home visit [18]. Despite the small number of subgroups characterized by underweight and obesity, statistically significant differences were found. The BMI was measured only at baseline, and self-reported weight at age 60 may be inaccurate due to recall bias. This value was reported by participants who could be affected by memory defects, causing inaccuracy. Furthermore, the cross-sectional design of this study precludes any causal inference. Finally, as this study only involved the population of Sardinia, the findings cannot be easily extended to long-lived populations of different ethnic origins. However, the homogeneity of the population under examination makes the results reliable and could provide a stimulus for similar studies in other long-lived populations.

## 5. Conclusions

This study indicates that mild overweight in nonagenarians is associated with extended lifespan. Older adults with this condition may have greater fat mass availability, which promotes resilience to the stressful events commonly experienced in old age, such as infectious diseases or fracture sequelae, and favors better performance in activities of daily living. Whatever mechanism the overweight paradox is based on, its impact suggests that the geriatrician should carefully consider the characteristics of excess fat, its distribution, and the concomitant reserve of lean mass when recommending effective dietary interventions in the elderly.

## Figures and Tables

**Table 1:** Background demographic characteristics of 200 elderly participants from the Sardinia Blue Zone.

Variables	Males	Females
No.	100	100
Age at recruitment (years)	92.6 ± 6.2	94.1 ± 5.0
Age range (years)	89–107	89–106
Number of participant alive at the end of follow-up	18	10
Mean survival time for 172 participants who died during the follow up (years)	2.83 ± 1.56	2.79 ± 1.55
Survival time for all participants (years)	3.25 ± 1.75	3.04 ± 1.67

**Table 2:** Median survival of study participants according to sex and BMI categories.

No. of Participants		BMI ^1^ Range (kg/m^2^)	Survival Time (years) ^2^	BMI ^1^ Range (kg/m^2^)	Survival Time (years) ^2^	*p*-Value for Males and Females Combined ^3^
Males	Females	Males		Females		
12	7	<18	1.80 [0.1;5.0]	<18	2.20 [1.1;2.6]	<0.0001
44	51	18.0–24.9	4.10 [0.1;6.0]	18.0–24.9	3.50 [0.3;6.6]	reference
19	19	25.0–27.0	5.11 [0.8;6.9]	25.0–27.2	4.01 [0.9;6.5]	0.038
18	19	27.1–29.9	2.80 [0.6;6.0]	27.3–29.9	2.30 [0.4;6.2]	0.037
7	4	≥30	2.70 [1.1;3.6]	≥30	2.10 [0.2;3.9]	0.014
Total 200			3.50 [0.1;6.9]		3.10 [0.2;6.6]	

^1^ BMI: body mass index; ^2^ median [range]; ^3^ Mann–Whitney *U* test.

**Table 3:** Age-adjusted mean values of waist and limb circumferences of study participants, stratified according to sex and BMI at baseline.

No. of Participants	BMI ^1^ at Baseline (kg/m^2^)	Age-Adjusted Waist Circumference (cm)	Age-Adjusted Arm Circumference (cm)	Age-Adjusted Calf Circumference (cm)
Males				
12	<18	99.15 ± 5.90	27.75 ± 3.30	36.75 ± 2.98
44	18.0–24.9	99.22 ± 7.74	28.67 ± 4.19	33.31 ± 2.47
19	25.0–27.0	101.65 ± 8.07	27.31 ± 3.20	33.81 ± 3.33
18	27.1–29.9	102.60 ± 10.69	30.88 ± 4.22	37.50 ± 4.63
7	≥30	104.25 ± 11.29	29.50 ± 7.68	32.67 ± 2.08
Females				
7	<18	99.38 ± 6.57	24.20 ± 1.09	27.40 ± 2.61
51	18.0–24.9	98.99 ± 6.03	25.36 ± 2.67	30.80 ± 3.76
19	25.0–27.2	101.65 ± 3.28	26.47 ± 2.77	31.26 ± 4.03
19	27.3–29.9	102.62 ± 10.69	27.92 ± 3.90	32.38 ± 3.66
4	≥30	103.40 ± 9.96	28.25 ± 3.86	34.25 ± 6.94

^1^ Body mass index.

**Table 4:** Cox regression analysis with survival as the outcome and BMI categories as predictors. Covariates: sex, age, and comorbidity (CIRS score).

Covariates	Full Model (HR ^1^ and 95% CI)
Male sex	1.075 (0.757–1.527)
Age at baseline	1.396 (1.323–1.473) **
Comorbidity (CIRS score)	0.898 (0.754–1.070)
BMI, range (kg/m^2^)	
<18	2.869 (1.693–4.862) **
18.0–24.9	reference
25.0–27.1	0.610 (0.395–0.944) *
27.0–29.9	1.505 (1.000–2.264) *
≥30	2.703 (1.417–5.157) **

^1^ Hazard ratio; * *p* < 0.05; ** *p* < 0.001.

**Table 5:** Association between food consumption and survival status of study participants.

Foods	Participants Dead during the Follow-Up (n = 172)	Participants Alive at the End of Follow-Up (n = 28)	Total (n = 200)
Beef meat	2.14	2.36	2.17
Goat/pork meat	2.01	1.67	1.97
Poultry meat	1.56	1.76	1.59
Fish	1.59	1.52	1.58
Legumes	3.71	3.92	3.74
Leafy greens	3.31	3.48	3.34
Fruit	3.55	4.08 *	3.62
Bread	4.77	4.96	4.80
Pasta	3.49	3.72	3.52
Potato	4.19	4.32	4.21
Olive oil	2.78	3.20 *	2.85
Lard	3.23	3.22	3.23
Sweet	2.29	2.16	2.27
Cheese	4.86	4.88	4.86
Milk	4.77	4.87	4.78
Coffee	3.64	3.78	3.66
Wine	4.08	4.17	4.09

* *p* < 0.05 (Mann–Whitney *U* test).

**Table 6:** Association between food consumption and the categories of BMI.

Foods	BMI ^1^ <18 kg/m^2^	BMI 18.0–24.9 kg/m^2^	BMI 25.0–27.1 kg/m^2^	BMI 27.2–29.9 kg/m^2^	BMI ≥30 kg/m^2^	χ^2^/*p*-Value ^2^
Beef meat	1.43	2.04	2.10	3.06	2.82	23.30/<0.0001
Goat/pork meat	1.71	1.92	1.95	2.50	2.00	5.09/0.278
Poultry meat	1.00	1.47	1.95	2.12	1.73	21.60/<0.0001
Fish	1.29	1.59	1.52	1.71	1.55	1.91/0.752
Legumes	4.14	3.71	3.76	3.71	3.82	0.79/0.940
Leafy greens	3.57	3.29	3.43	3.35	3.45	2.54/0.637
Fruit	3.86	3.45	4.14	3.94	3.73	9.45/0.051
Bread	5.00	4.77	4.86	4.88	4.73	4.00/0.406
Pasta	3.29	3.53	3.67	3.41	3.45	4.35/0.361
Potato	4.71	4.11	4.38	4.35	4.36	5.06/0.281
Olive oil	2.71	2.87	2.86	2.76	2.82	0.26/0.992
Lard	3.57	3.25	3.32	2.75	3.33	6.49/0.165
Sweet	2.71	2.30	2.14	2.18	2.09	4.61/0.329
Cheese	4.86	4.84	4.86	4.94	4.91	0.51/0.973
Milk	5.00	4.81	4.86	4.53	4.64	4.28/0.369
Coffee	3.57	3.65	3.63	3.75	3.83	0.18/0.996
Wine	3.71	4.13	4.05	3.82	4.50	3.54/0.471

^1^ BMI: body mass index; ^2^ Kruskal–Wallis non-parametric test.

## Data Availability

Data are contained within the article and Supplementary Materials. The data will be available upon reasonable request.

## Supplementary Materials

The following supporting information can be downloaded at: https://www.mdpi.com/article/10.3390/jcm13175322/s1, Table S1: Survival of 113 study participants who provided their body weight at age 60. Supplementary Table S2: Mean values of ADL and IADL and comorbidity (CIRS score) of study participants stratified by BMI at baseline. Supplementary Table S3: Comorbidity among study participants according to the weight categories. Figure S1: Correlation between BMI at baseline and BMI at age 60.
