# Supplementary material for: Association between Mild Overweight and Survival: A Study of an Exceptionally Long-Lived Population in the Sardinian Blue Zone
Source: J Clin Med. 2024 Sep 9;13(17):5322. doi: 10.3390/jcm13175322 (PMC11396680; doi:10.3390/jcm13175322)
Supplement: Supplementary file 1 [file jcm-13-05322-s001.zip › jcm-3170192-supplementary.pdf]

Supplementary Figure S1 illustrates the correlation between BMI at baseline and BMI at age 60. The correlation was relatively high (r=0.337), indicating that those who were overweight or obese at baseline were likely to have been so during most of their adult life (**Supplementary Figure S1**).

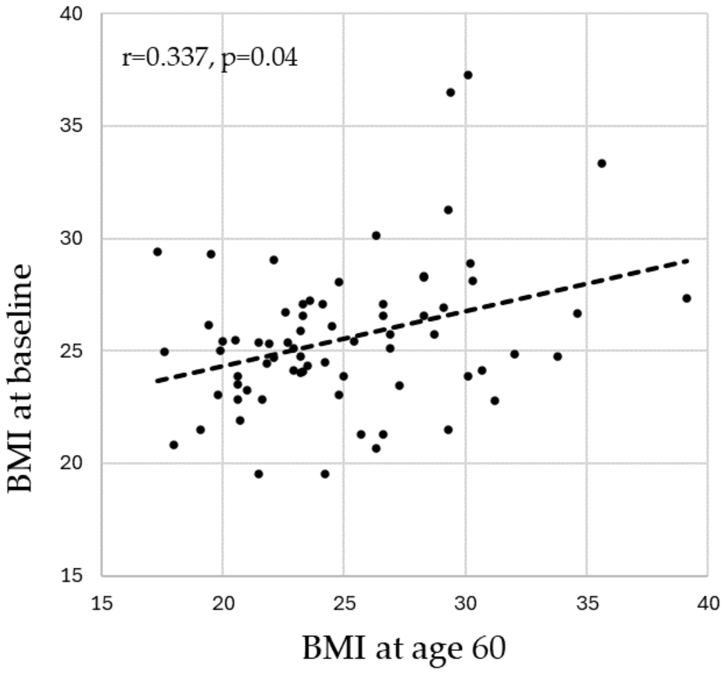

Figure S1. Correlation between BMI at baseline and BMI at age 60.

Supplementary Table S1 reports age at recruitment and survival in years, stratified by BMI value at age 60 for 113 out of 200 subjects (56%). Survival was longer, although non significantly, in subjects who were mildly overweight at age 60 years compared to their underweight or obese counterparts.

Supplementary Table S1. Survival of 113 study participants who provided their body weight at age 60.

No. of participants		BMI at age 60 (kg/m^2)	BMI mean (kg/m^2)	Mean survival time (years)		*p*-value for males and females combined
Males	Females			Males	Females	
1	12	< 18	–	4.90 [1]	2.42 ± 0.50	reference
35	22	18.0 – 24.9	23.11 ± 11.56	3.83 ± 1.72	3.41 ± 1.78	0.380
13	9	25.0 – 26.9	24.92 ± 1.61	4.02 ± 1.97	5.13 ± 1.35	0.095
10	3	27.0 – 29.9	28.75 ± 0.51	3.90 ± 2.00	4.03 ± 0.32	0.880
4	6	≥ 30	32.51 ± 2.91	3.14 ± 2.30	0.85 ± 0.33	0.912
Total 113			23.77 ± 3.79	3.71 ± 2.13	3.17 ± 1.75	

[1] The standard deviation cannot be calculated because there was only one participant.

Supplementary Table S2. Mean values of ADL and IADL and comorbidity (CIRS score) of study participants stratified by BMI at baseline.

No. of participants	BMI [1] at baseline (kg/m²)	ADL score	IADL score	CIRS score
19	< 18	2.5 ± 1.7	1.9 ± 1.2	2.6 ± 0.9
95	18.0 – 24.9	3.9 ± 1.6	4.6 ± 3.9	2.0 ± 1.0
48	25.0 – 26.9	3.6 ± 1.5	3.9 ± 3.4	1.9 ± 1.1
27	27.0 – 29.9	4.5 ± 1.4	4.4 ± 3.1	1.4 ± 1.1
11	≥ 30	5.2 ± 0.9 *	7.9 ± 4.3 *	1.7 ± 1.3

[1] Body mass index; The category of BMI 25.0–26.9 was selected as the reference group; * $p<0.05$

Supplementary Table S3. Comorbidity among study participants according to the weight categories.

**Illnesses**	**BMI [1] (kg/m²)**			
	**< 18**	**18.0 – 24.9**	**25.0 – 27.1**	**≥ 27.2**
No. of participants	19	95	48	38
Cardiac	4 (21.0%)	9 (9.5%)	3 (6.2%)	7 (18.4%)
Vascular	1 (5.3%)	1 (1.0%)	1 (2.1%)	1 (2.6%)
Hematological	0	0	0	0
Respiratory	0	1 (1.0%)	1 (2.1%)	2 (5.3%)
Ophtalmological and ORL	5 (26.3%)	9 (9.5%)	4 (8.3%)	6 (15.8%)
Upper gastrointestinal	1 (5.3%)	1 (1.0%)	0	1 (2.6%)
Lower Gastrointestinal	1 (5.3%)	0	0	1 (2.6%)
Hepatic and pancreatic	1 (5.3%)	1 (1.0%)	0	1 (2.6%)
Renal	0	0	0	0
Genitourinary [2]	4 (21.0%)	6 (6.3%)	3 (6.2%)	2 (5.3%)
Muscoloskeletal and tegumental	5 (26.3%)	11 (11.6%)	5 (10.4%)	6 (15.8%)
Neurological	2 (10.5%)	2 (2.1%)	2 (4.2%)	3 (7.9%)
Endocrine, metabolic	1 (5.3%)	1 (1.0%)	1 (2.1%)	6 (15.8%)
Breast	0	1 (1.0%)	0	0
Psychiatric	1 (5.3%)	1 (1.0%)	0	0

[1] Body mass index; [2] including cancer

Supplemental Table S2 illustrates the illnesses identified through the CIRS questionnaire. The frequency of diabetes (endocrine and metabolic diseases) was higher among the participants with obesity. The frequency of the other illnesses was comparable or lower in the mild overweight, category.
